# Orthopaedic Trauma Surgeons' Financial Relationships With Industry: An Analysis of the Sunshine Act Reporting of Physician Open Payments From 2014 to 2019

**DOI:** 10.5435/JAAOSGlobal-D-21-00251

**Published:** 2021-11-09

**Authors:** Nicholas Frane, Matthew J. Partan, Peter B. White, Cesar Iturriaga, John M. Tarazi, Trinava Roy, Adam D. Bitterman

**Affiliations:** From the The Center for Orthopedic Research and Education (CORE) Institute, Orthopaedic Trauma Fellowship, Phoenix, AZ (Frane); the Northwell Health—Huntington Hospital, Department of Orthopaedic Surgery, Huntington, NY (Partan, White, Iturriaga, Tarazi, and Bitterman); Donald and Barbara Zucker School of Medicine at Hofstra/Northwell, Huntington, NY (Partan, White, Iturriaga, Tarazi, and Bitterman); and the Rowan University School of Osteopathic Medicine, Stratford, NJ (Roy).

## Abstract

Orthopaedic trauma surgeons have an intricate relationship with the medical device industry. In the past decade, legislation has created transparency of monetary exchanges between physicians and industry. In 2013, the Physician Payments Sunshine Act was passed and ultimately led to the creation of the Open Payments Database. The purpose of this study was to evaluate trends in industry payments to orthopaedic trauma surgeons.

**Methods:**

A retrospective review of the Centers for Medicare & Medicaid Services' Open Payments Database was conducted for general industry payments to orthopaedic trauma surgeons from 2014 to 2019. Total payments and subtype payments were analyzed for yearly trends. All payments were converted to 2019 US dollars to adjust for inflation. Descriptive statistics included analysis of payments, number of surgeons, types of payments, top contributing companies, and regional comparisons. Trends were assessed through the Jonckheere-Terpstra test. Statistical significance was defined at *P* < 0.05.

**Results:**

From 2014 to 2019, 45,312 individual payments were given to orthopaedic trauma surgeons (N = 3208) accounting for a total of $41,376,397.85 (USD), with a mean of $919.54 per payment. Increased trends were noted for median annual payments, number of payments, and number of surgeons receiving payments. Compared with 2014 ($460.91), median payments were increased by 90.9% in 2016 ($879.85), 102.6% in 2018 ($933.81), and 178.6% in 2019 ($1284.06). Payment subtypes that demonstrated increasing median payments included consulting fees (*P* = 0.028); education (*P* < 0.001); entertainment, food, and beverage (*P* < 0.001); and travel (*P* = 0.019). Decreases in median payments were seen in royalties (*P* = 0.044) and grant funding (*P* < 0.001). Regional comparisons demonstrated increasing trends in median payments in the midwest (*P* = 0.011), south (*P* < 0.001), and west (*P* = 0.003), but not in the northeast (*P* = 0.081).

**Discussion:**

In our study, we found that industry payments to orthopaedic trauma surgeons were increasing markedly between 2014 and 2019, particularly among consulting fees, education, entertainment, food and beverage, and travel.

As part of the US Patient Protection and Affordable Care Act in 2010, the Physician Payments Sunshine Act (PPSA) required drug, device, biological, and medical supply manufactures to report any monetary transfer greater than $10 (US dollars) to physicians to the Centers for Medicare & Medicaid Services (CMS).^1^ Although manufactures have a history of financial arrangements with physicians, the magnitude of the financial exchanges has largely been obscured to the public.^2-5^ In recent times, there have been increasing concerns regarding conflicts of interest and their implications on medical decision making.^6,7^ Consequently, CMS started the National Physician Payment Transparency Program that developed the Open Payments Database (OPD), a publicly available resource disclosing financial exchanges between physicians and industry. Supporters of the legislation saw this as a pivotal move to strengthen trust in the medical profession. However, it was met with controversy and seen by others as an attempt to deter physicians from accepting payments.^8,9^ In total, manufacturers and group purchasing organizations have reported 76.25 million recorded payments accounting for roughly $53 billion in payments to physicians and teaching hospitals.^10^ Previous studies have shown that orthopaedic surgeons receive the highest average payment when compared with other specialties.^11^

The role of physician involvement in product development and industry success has been substantial over the past century. The history of orthopaedic implant evolution exemplifies the integral role of orthopaedic trauma surgeons in innovation and development.^12,13^ For example, a Swiss study group founded Arbeitsgemeinschaft für Osteosynthesefragen, the Association for the Study of Internal Fixation, with the intentions to expand on the infancy of orthopaedic implant pioneering and treatment of patients with fractures. In the present day, the Arbeitsgemeinschaft für Osteosynthesefragen foundation has maintained a symbiotic relationship with modern-day industry to fund continued education and research.^14^ The financial benefit of osteosynthesis was highlighted by a recent publication demonstrating a cost savings of over a trillion dollars, solely from osteosynthesis (femur, tibia, and radius) over the past 60 years.^15^ Similarly, physician relationships with industry have proven to be integral on advancing care across many medical and surgical subspecialties. However, not all financial interactions are judicious. The legislation put forth in the PPSA was to make all financial relationships transparent, so the public could interpret which payments have potential for implicit bias. The large variety in payment types, which range from representatives paying for lunch, educational courses, consulting services, and physician royalties for implant development, further complicates interpretation of these payments.

The collegial relationship between orthopaedic trauma surgeons and the medical device industry is well established; however, the effects of the aforementioned PPSA legislation have yet to be examined in this population of physicians. In addition, there has been a paucity of orthopaedic literature examining the trends across multiple years and subtypes of payments. The purpose of this study was to evaluate trends in industry payments to orthopaedic trauma surgeons from 2014 to 2019. We hypothesized that increased transparency, created by the mandated OPD reporting, would result in decreasing payments to surgeons across the study period. Secondary outcomes included evaluating regional differences in payments and yearly trends in subpayment categories. Finally, we sought to determine the top industry companies providing payments to orthopaedic trauma surgeons.

## Methods

A retrospective review of the CMS OPD was done to identify all payments to trauma-trained orthopaedic surgeons between 2014 and 2019. The CMS OPD includes all physicians from the United States who received at least one industry payment or transfer of value worth at least $10. The OPD has released six data sets including the last five months of 2013 and the full years of 2014 to 2019. For the purpose of this study, we excluded the data from 2013 to avoid seasonal and confounding variables.^16-18^

To conduct this study, the data set was stratified to identify all payments to orthopaedic surgeons (n = 1,941,772). The data set was then further stratified based on orthopaedic subspecialty by selecting payments specialty “Allopathic & Osteopathic Physicians|Orthopaedic Surgery|Orthopaedic Trauma” and then combining payments by identification number and year. Orthopaedic trauma surgeons were identified and aggregated by individual years and treated as independent samples for yearly comparisons. A total of 3,208 orthopaedic trauma surgeons received at least one payment over the study period. Analysis of the publicly available OPD contained no protected health information and did not require Institutional Review Board review at the authors' institution.

General payment subtypes including charitable contributions, faculty or speaking fees, consulting fees, ownership or investment payments, educational payments, entertainment/food and beverage payments, gifts, grants, honoraria, royalties, and travel were collected.^8^

The primary outcome of the study was to determine whether there is a trend in total overall general payments per surgeon from 2014 to 2019. Secondary outcomes included analyzing yearly payment trends to orthopaedic trauma surgeons across subtype and regions. Regions were created according to the US census regions (northeast, south, midwest, and west).

Total payments and subtype payments (nature of payment) were calculated as yearly aggregates. Descriptive statistics were calculated for the years 2014 to 2019 to analyze the number of payments, number of surgeons receiving a payment each year, nature of payments, top contributing companies, and regional comparisons. Industry contributions were also calculated annually to determine the top five contributing companies. All payments were adjusted to the 2019 Consumer Price Index to account for inflation between years.^19^

To evaluate regional data, orthopaedic trauma surgeons were grouped based on US census regions. The median general payment per surgeon and interquartile range for each census region were also calculated by year. For each US state and the District of Columbia, sum payments for all years and yearly median total payments to surgeons per state were calculated and used to make choropleth maps.

For total payments and subtype payments, we did the Shapiro-Wilk test, which demonstrated that the data were not distributed normally (*P* < 0.001). Yearly trends were assessed through the nonparametric Jonckheere-Terpstra test for ordered alternatives to account for increased payment trends, which has more statistical power compared with the Kruskal-Wallis test when there is an a priori ordering.^20^ To compare year to year, we also defined the first full year of data (2014) as our index year and compared each individual year (2015 to 2019) against the index year using the Mann-Whitney *U* test. A Spearman rank order correlation was performed to assess the relationship between years and total number of payments, total number of surgeons, and yearly payments per surgeon. All statistical testing was done with SPSS version 26 (IBM) and an alpha value of 0.05.

## Results

### Annual Trends in Payments

Throughout the 6-year period, 45,312 individual payments were given to orthopaedic trauma surgeons (N = 3,208) for a total of $41,376,397.85 (USD), with a mean of $919.54 per payment. Annual total payments, number of payments, number of surgeons, and mean payment amount are further summarized in Table 1.

**Table 1:** Total Trends for Increasing Yearly Payments

Year	Sum Payments ($)	No. of Payments	Mean Payment ($)	Surgeons (N)	Median Yearly Payment (25th-75th Quartiles), $	*P* ^a^
2014	$5,953,864.34	6316	$942.66	509	$460.91 (104.17-2731.80)	—
2015	$5,886,575.49	6592	$892.99	519	$517.21 (141.88-3150.41)	1.000^a^
2016	$8,124,671.86	6663	$1219.37	495	$ 879.85 (144.12-4635.37)	0.030^a,b^
2017	$5,416,303.52	6622	$817.93	546	$ 455.93 (118.40-2856.39)	1.000^a^
2018	$8,463,776.15	8680	$975.09	555	$ 933.81 (161.42-5660.90)	<0.001^a,b^
2019	$7,531,206.50	10,124	$743.90	584	$1284.06 (202.03-5557.73)	<0.001^a,b^

The Jonckheere-Terpstra test for ordered alternatives demonstrated increase median yearly payment with increasing years (*P* < 0.001).

a Statistical significance reached.

b The pairwise Mann-Whitney *U* test compared with 2014.

When evaluating median total payments per surgeon, a significant positive trend was found with increasing years across the study period (*P* < 0.001). When compared with 2014 ($460.91), significant increases were observed in median payments by 90.9% in 2016 ($879.85), 102.6% in 2018 ($933.81), and 178.6% in 2019 ($1,284.06) (Table 1).

Throughout the study period, the total number of payments increased to 60.3% (r = 0.943; *P* = 0.005) and were distributed among a 14.7% increased number of surgeons (r = 0.829; *P* = 0.042). The total payment per surgeon positively correlated with years in the study period (r = 0.107; *P* < 0.001) (Table 1).

Between 2014 and 2019, 82.6% (N = 2648) of the orthopaedic trauma surgeons receiving payments acquired a yearly total payment of less than $10,000, which made up 8.2% of the total industry payment sum. Those receiving a yearly total payment greater than $500,000 accounted for 0.2% of surgeons but received 14.98% of the sum payments (Table 2).

**Table 2:** Distribution of Yearly Industry Payments to Orthopaedic Trauma Surgeons

Yearly Total Payment ($)	% of Sum Contributions	Surgeons (N)	% of Total Surgeons
<$100	0.1	622	19.4
$100-<$1000	1.0	1128	35.2
$1000-<$10,000	7.1	898	28.0
$10,000-<$100,000	39.0	473	14.7
$100,000-<$500,000	38.0	80	2.5
≥$500,000	14.9	7	0.2

Yearly total payment per year including 2014–2019 open payments data.

### Comparison of Payment Subtypes

**Figure 1:**
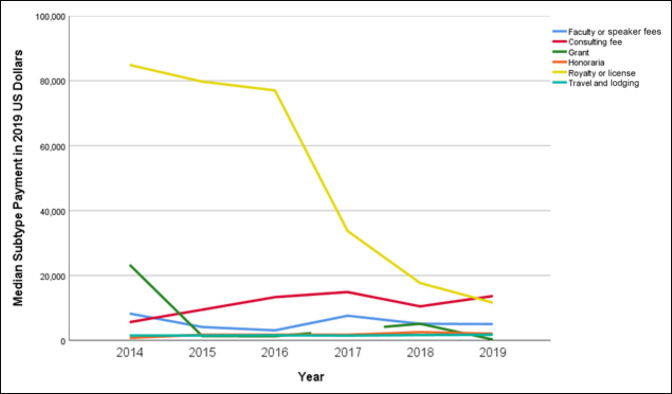
Chart showing median payments by subtype per year.

**Figure 2:**
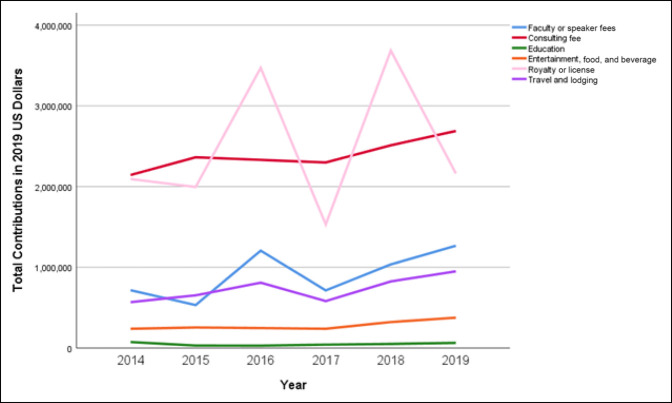
Chart showing total contributions by payment subtype per year.

When evaluating subtypes of payments, increasing median payment trends were observed in consulting fees (143.8%, *P* = 0.028); education (261.1%, *P* < 0.001); entertainment, food, and beverage (88.2%, *P* <0.001); and travel (15.5%, *P* = 0.019; *P* = 0.001; Table 3). From 2014 to 2019, median payments showed a downward trend for faculty or speaker fees (39.5%, *P* = 0.104), grants (98.9%, *P* < 0.001), and royalty or license (86.3%, *P* = 0.044) (Figure 1). The number of orthopaedic trauma surgeons receiving contributions because of royalty and licensing increased by 107% from 14 in 2014 to 29 in 2019. The sum of contributions involving royalties and licensing had large variations from year to year, whereas the consulting fees had consistent growth corresponding to a 25.4% increase (Figure 2). The highest sum payments were characterized as royalty or license fees making up $14,939,439.48 (36.1%), followed by consulting fees $14,336,897.20 (34.6%)and faculty or speaker fees $5,472,012.01 (13.2%).

**Table 3:** Subtype Payment Aggregates Based on Median Payment per Surgeon

Median (IQR) ($)							*P*
Payment Subtype	2014	2015	2016	2017	2018	2019	
Faculty or speaker fees	8262.00 (22,086.00)	4098.32 (13,554.94)	3061.88 (7,210.31)	7587.825 (12,489.92)	5090 (7,635.00)	5000.00 (6,254.69)	0.104
Consulting fee	5597.87 (20,295.36)	9440.45 (2,4801.08)	13,307.22 (32,028.61)	14,872.62 (30,763.48)	10,466.85 (26,595.25)	13,650.00 (28,625.00)	0.028^a^
Education	102.61 (304.452)	106.82 (367.03)	255.59 (1340.01)	423.33 (1578.79)	641.34 (1503.08)	370.49 (1,334.50)	<0.001^a^
Entertainment, food, and beverage	217.78 (549.27)	248.75 (502.15)	284.80 (594.47)	240.04 (495.37)	303.97 (549.37)	409.86 (749.55)	<0.001^a^
Grant	23,287.50 (29,632.50)	1294.80 (2,077.08)	1278.00 (2,213.23)	—	5,090.00 (0.00)	250.00 (200.00)	<0.001^a^
Honoraria	702.00 (2,106.00)	1735.03 (867.52)	1721.04 (860.52)	1718.86 (859.43)	2516.496 (989.50)	2055.00 (1,411.00)	0.149
Royalty or license	84,863.53 (16,1131.10)	7,9730.72 (196,898.09)	77,036.09 (243,540.12)	33,707.04 (101,343.05)	17,656.22 (165,051.20)	11,605.25 (67,988.29)	0.044^a^
Travel and lodging	1491.84 (2,642.26)	1489.46 (2,056.71)	1552.34 (2,901.12)	1446.25 (2,293.28)	1636.38 (2,974.02)	1723.55 (2,677.26)	0.019^a^

IQR = interquartile range

a The Jonckheere-Terpstra test for ordered alternatives done with significance defined as *P* < 0.05.

### Regional Comparisons

**Figure 3:**
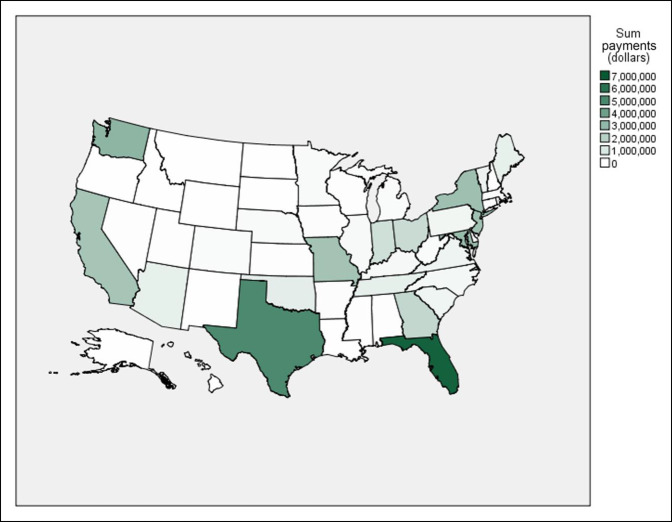
Choropleth showing sum payments for all years by state.

**Figure 4:**
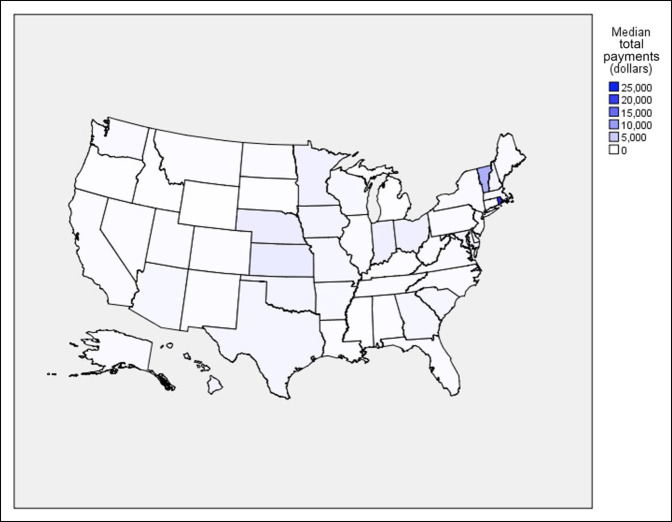
Choropleth showing median yearly total payments per surgeon by state.

Across the study period, regional comparisons demonstrated upward trends in median payments in the midwest (163.4%, *P* = 0.024), south (208.3%, *P* < 0.001), and west (102.3%, *P* = 0.009), but not in the northeast (*P* = 0.081). Nevertheless, the northeast had a 3.8-fold increase in median payments between 2014 and 2019 ($326.90 versus $1683.62, *P* = 0.001). Across the 6 years, Florida was the recipient of the largest sum contributions (total sum: $6,979,706.61; 16.9%; number of surgeons: 263; 8.2%; and yearly median payment per surgeon: $405.18) while Rhode Island had the largest yearly median payments per surgeon (total sum: $273,406.58; 0.7%; number of surgeons: 12; 0.4%; and yearly median payment: $23,156.44) (Figures 3 and 4).

### Top Companies

**Figure 5:**
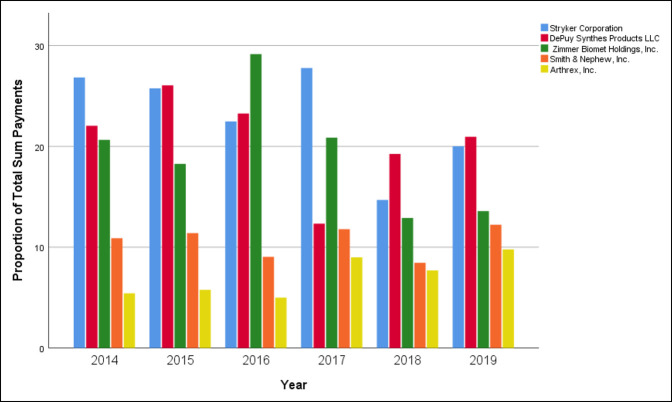
Chart showing sum of the top five companies per year.

Total payment sums were calculated based on the contributing company, LLC. The top five companies contributed a total of $32,993,271.61 between 2014 and 2019, which constituted 79.7% of total payments to orthopaedic trauma surgeons. Stryker had the highest total payments which totaled $9,195,263.39; 22.2% of total contributions, followed by DePuy Synthes ($8,610,968.02; 20.8%), Zimmer Biomet Holdings ($7,917,019.07; 19.1%), Smith and Nephew ($4,327,935.81; 10.5%), and Arthrex ($2,942,085.31; 7.1%). Individual yearly comparisons can be seen in Figure 5.

## Discussion

Research is beginning to provide clarity on the evolving landscape of physician-industry relationships.^21^ Increased transparency, through the OPD, has allowed individuals to draw invaluable associations between rising pharmaceutical industry payments to physicians. Previous studies have demonstrated that pharmaceutical payments lead to greater prescribing of opioids, expensive cancer treatment therapies, branded medications, and prescribing costs per patient.^22-24^ These relationships potentially affect physician medical decision making at the expense of patients. Although these concerns are relevant for orthopaedic surgeons, the medical device industry provided most funding in our study. Given their clinical and surgical expertise, orthopaedic surgeons are in a unique position to work in concert with industry to advance innovation and improve patient care. Surgeons who improve patient care through this avenue should be incentivized for their contributions. In comparison with other specialties, orthopaedic surgery has been highlighted as receiving the highest payments, despite representing a small percentage of total physicians nationally (2.87%).^11,25^ This study is the first to give an inclusive trend analysis using 6 years of industry payments to orthopaedic trauma surgeons.

The authors found that industry payments to orthopaedic trauma surgeons increased between 2014 and 2019. A notable positive trend in median payments per surgeon was found with increasing years across the study period. In comparison with 2014, the median payment per surgeon almost tripled from $426.77 to $1284.06 in 2019. Furthermore, an increase was observed in both the total number of payments and number of surgeons receiving payments. The total payment per surgeon positively correlated with years in the study period. The PPSA, along with the accompanying media, has painted a pernicious picture surrounding these financial relationships.^2^ We hypothesized that payments would decrease based on the theory that physicians, specifically orthopaedic trauma surgeons, would be deterred by the law; this was ultimately not consistent with our findings. One of the outliers in the data was the 2017 reporting year, where total payments decreased 33.3% and median payments decreased 48.2%. Most of these decreases were payments for royalties and grants. We were unable to find any regulatory changes with reporting that could account for these changes in the literature. However, it seems the decrease was not unique to orthopaedic trauma surgeons. Total payments to all physicians, irrespective of specialty, decreased by 10% from the previous year, and the largest decrease was seen in payments for grants.

Trend analysis of payment subtypes demonstrated that median payments for royalties did not increase between 2014 and 2019 (*P* = 0.062). Median payments for royalties remained stable above $72,000 in 2014 to 2016 and decreased to $32,317.40 (2017), $17,344.03 (2018), and $11,605.25 (2019). Conversely, total royalty amounts were shown to increase over the study period. Additional analysis determined that the number of surgeons receiving royalties doubled from 2014 to 2019. Despite median payments decreasing, one could make the inference that surgeons are becoming more involved with product development and hence the increase in total royalty payments and number of surgeons receiving payments. Consulting fees, on the other hand, showed increasing trends in median payment per surgeon over the study period. Payments for education demonstrated similar increases in median payments and total payments. These findings suggest that increased transparency has not deterred the cultivation of industry relationships, but rather persisted by supporting innovation in the field.

Previous studies analyzing the OPD demonstrated varying trends among physicians in different specialties. For example, a study looking at medical oncologists found that the number of physicians receiving payments has been declining since 2014.^26^ The authors suggested that physicians may be less likely to engage in financial relationships with industry because of the increased transparency and public perception. Similar trends were seen in a study evaluating payments to plastic surgeons.^27^ The same study also found an associated reduction in total dollars related to payments.^27^ By contrast, this study demonstrated increasing trends in the number of orthopaedic trauma surgeons, number of payments, and median payments per surgeon from 2014 to 2019. Previous work has looked at orthopaedic surgeons' evolving relationship with industry. Specifically, Pathak et al^28^ found that median payments for orthopaedic spine and foot and ankle surgeons failed to have notable changes between 2014 and 2017. Similar findings have been reported for reconstruction-trained orthopaedic surgeons.^29^ In contrast to these studies, the findings in this study demonstrate increasing trends in payments. It is difficult to account for the exact differences in trends. Accounting for the differences in trends is difficult to elucidate; however, our analysis demonstrated trends that were not previously captured because previous studies had shorter study periods. Orthopaedic trauma is heavily tied to industry through education, innovation, and evolution of the field,^12^ which may in part explain the continued growth of industry funding to orthopaedic trauma surgeons.

Physicians who play influential roles in industry development seem to be the largest benefactors from these financial relationships. In this study, the large variation in the data, demonstrated by the large interquartile ranges, suggests a large percentage of industry payments were made to a small percentage of trauma surgeons. Specifically, the top 2.7% (N = 87) of trauma surgeons were paid 52.9% of the sum payments. Furthermore, 19.4% (N = 622) of surgeons received less than $100.00 annually, which made up 0.1% of the total sum of financial contributions. Similar variations were seen in a recent study by Partan et al^30^ which found that among sports medicine orthopaedic surgeons, the top five compensated surgeons received 45.8% of all industry contributions. Most of these payments were from physicians receiving royalties. In addition, 89.4% of the total sports medicine surgeons received yearly total payments of less than $10,000.00. Collectively, the relationships between industry and high-earning physicians seem to be minimally affected by the PPSA.^31-33^ Because most of these physicians are receiving royalties for product development and innovation, they are likely more resistant to changes in trends from year to year.

A paucity of data is present demonstrating the difference regionally in industry payments to physicians. Previous research has failed to illustrate clear trends in median payments in any geographic regions.^28,29^ By contrast, our study shows that median payments in the midwest (*P* = 0.011), south (*P* < 0.001), and west (*P* = 0.003) had significant upward trends. Garstka et al^11^ determined that industry payments were markedly different in particular states in comparison with others and suggested that state government regulation has played a role in this distribution. Similarly, Wills et al^34^ did a study on orthopaedic surgery residents receiving industry payments, showing that residents in least restrictive states were more likely to receive payments than those in more restrictive states. We believe that additional research is needed to determine the factors contributing to the geographic distribution of reported industry payments.

The present study has limitations. First, the results of our analysis are limited by the inclusiveness and accuracy of the OPD data submitted to CMS. In the inaugural year of OPD publication, the CMS redacted more than 40% of the payment records submitted secondary to major inaccuracies.^18,35^ However, the US Department of Health and Human Services conducted a review of accuracy, precision, and consistency in reporting of the 2015 OPD and found that less than 1% were missing mandatory data elements.^17^ Second, only applicable manufactures that receive payments from Medicare, Medicaid, or the Children's Health Insurance Program are included in the OPD. However, the authors believe that the volume of data in the OPD is an accurate representation of industry trends across years. Third, the data are submitted by industry, which inherently puts it as risk for selection and reporting bias. Fourth, the available data only include published data, which fail to take into account unidentified records; or records withheld secondary to specific manufacture requests, delays in publication, and unresolved disputes by physicians; or failed submissions for covered recipients by manufacturers and group purchasing organizations. Despite these limitations, the OPD is the largest data set of industry physician relationships presently available.

The current analysis is the first of its kind, examining payments to orthopaedic trauma surgeons. In addition, previous research has lacked a comprehensive six-year evaluation of the OPD. In addition, the utility of the nonparametric Jonckheere-Terpstra test trend analysis strengthens the power and accuracy of assessing trends in a nominal direction when applied priori.^20^ This study strongly suggests that the industry-physician relationship among orthopaedic trauma surgeons has expanded in the face of transparency, with median payments, total payment amount, and number of payments between 2014 and 2019 trending up.

Identifying the distribution of these financial exchanges is the first step to understanding how these relationships affect patient care, costs, and advancement in the field of orthopaedics. The authors believe that close relationships with industry have contributed to pivotal advancements in the field of orthopaedics and are essential for innovation. Future research should aim to determine whether any of these financial relationships affect the cost of care to patients and treatment equipoise. This study engendered thought-provoking concerns to the authors, namely whether the Sunshine Act and the OPD are succeeding with their goals and intentions. Political figures were quick to lionize the passing of the bill and implementation of the database commonly citing the importance of transparency to patients. In a recent longitudinal national survey, Kanter et al^36^ compared patient awareness of industry payments with physicians between 2014 (before the public release of the OPD) and 2016. The authors found that patients' awareness that industry payments information was publicly available only increased 3.2% (9.8% in 2014 versus. 12.9% in 2016). Furthermore, in 2016, 3.1% of patients knew whether their own doctor has received industry payments. They concluded that the current OPD was limited in its ability to inform patients. Further confounding this ethical conundrum, Iyer et al^37^ demonstrated that 88.8% of patients were unaware of their spine surgeon's relationship with industry, and 90.7% were unaware of the existence of the OPD. Irrespective of this, more than half of the patients indicated that surgeon-industry relationships are an important consideration when choosing their surgeon. In our opinion, it would behoove physicians, researchers, and political organizations to disambiguate the utility of this information: What payments actually influence decision making or patient perception and how these differ between recipients to effectively decrease costs and efficiency? As a result of the SUPPORT Act's expansion of “covered recipients,” the CMS will be requiring physician assistants, nurse practitioners, clinical nurse specialists, nurse anesthetists, and nurse midwives to be included in the OPD.^38^ In addition, they will be requiring three new payment categories: debt forgiveness, long-term medical supply or device loan, and acquisitions. The estimated burden of these requirements is just more than 1 million hours annually.^38^ Finally, an important takeaway of our study is to improve awareness and educate physicians on the OPD. The implications of false or inaccurate reports can be exorbitant and maintaining accuracy of their payments from industry should be monitored.
